# Supplementary material for: Unraveling the Molecular Mechanisms Underlying Spontaneous Multipolar Mitosis Through CIN‐seq
Source: Adv Sci (Weinh). 2026 Apr 27;13(40):e14238. doi: 10.1002/advs.202514238 (PMC13325660; doi:10.1002/advs.202514238)
Supplement: Supplementary file 1 — Supporting File: advs74401‐sup‐0001‐SuppMat.docx. [file ADVS-13-e14238-s001.docx]

Supporting Information

**Characteristics of MCF10A multipolar mitosis cells**

We observed a high survival rate among TP cells (99.4%, defined as surviving for > 24 hr or until the next mitosis). Among the surviving, mitotic TP cells, 63.2% continued with tripolar mitosis, while 36.8% reverted to bipolar mitosis (Figure S4a-ii and S4b; Figure S5). These results suggest the phenotypic plasticity of TP cells, as evidenced by their successful survival in the population and their ability to transition between mitosis types over generations.

Regarding the mother cells of TP cells, the majority of TP cells originated from mother cells undergoing bipolar mitosis (74.1%) (Figure S4a-ii), while others originated from mother cells undergoing tripolar/multipolar mitosis (25.9%) (Figure S4a-ii). Notably, 50.3% of TP cells originated from binucleate cells, where two nuclei merged or fused within a single cell (Figure S4c, S4d). This phenomenon may be attributed to cytokinesis failure or segregation errors over time.

Furthermore, compared with TP cells, the survival rate of cells undergoing tetrapolar mitosis is approximately 80% (16 of 20 identified cells survived), which is lower than that of TP cells. Figure S4e shows two representative cases of tetrapolar mitosis over time. This difference is reasonable, as tetrapolar mitosis often leads to more severe chromosomal mis-segregation and greater genomic instability than tripolar mitosis. With four spindle poles, chromosomes are distributed even more unevenly, resulting in extensive aneuploidy, chromosome loss, and DNA damage.

**Supporting Figures**

**Figure S1. GTWeka cell segmentation.** Real-time cell segmentation within CIN-seq is achieved through the ground-truth assisted trainable Weka segmentation (GTWeka) method, integrated within the CIN-seq pipeline. Scale bars denote 50 μm.


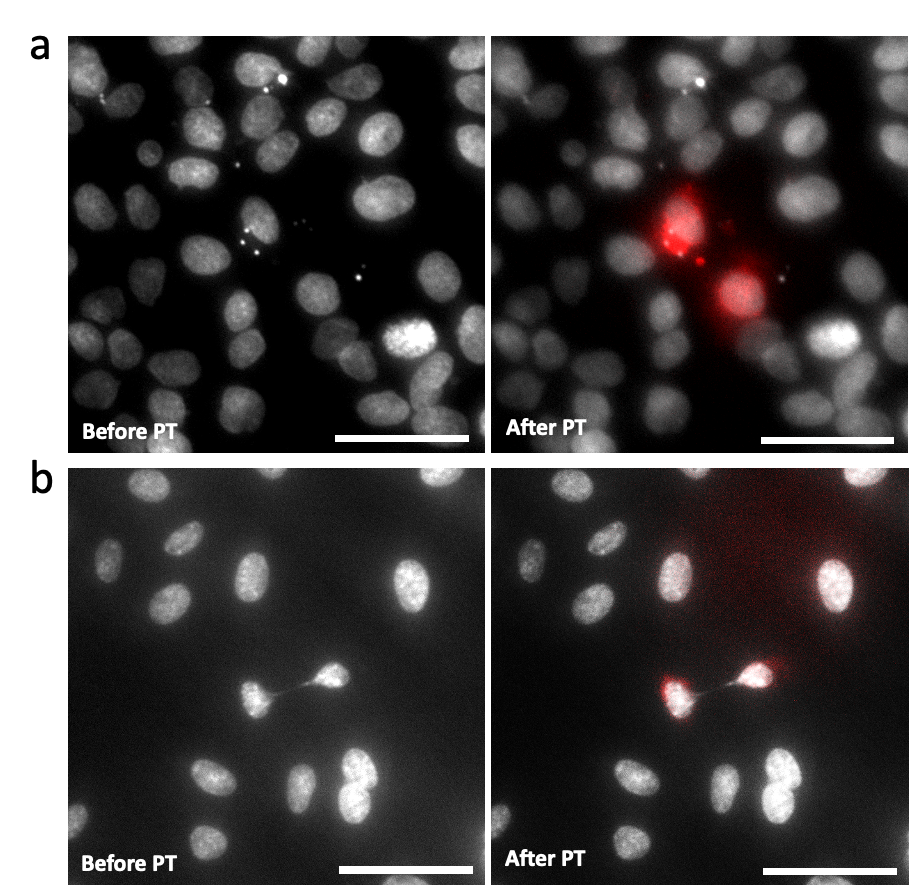


**Figure S2. Additional CIN phenotypes.** a) A representative image shows two instances of micronucleated (MN) cells, products of lagging chromosomes, from patient-derived esophageal adenocarcinoma (EAC) cells stained with SPY650-DNA nuclear dyes. Left panel: the image before phototagging (PT). Right panel: the image after PT (red). b) A representative image shows one instance of a chromatin bridge cell from patient-derived EAC cells stained with SPY650-DNA nuclear dyes. Left panel: the image before PT. Right panel: the image after PT (red). The scale bars denote 100 µm.

­­


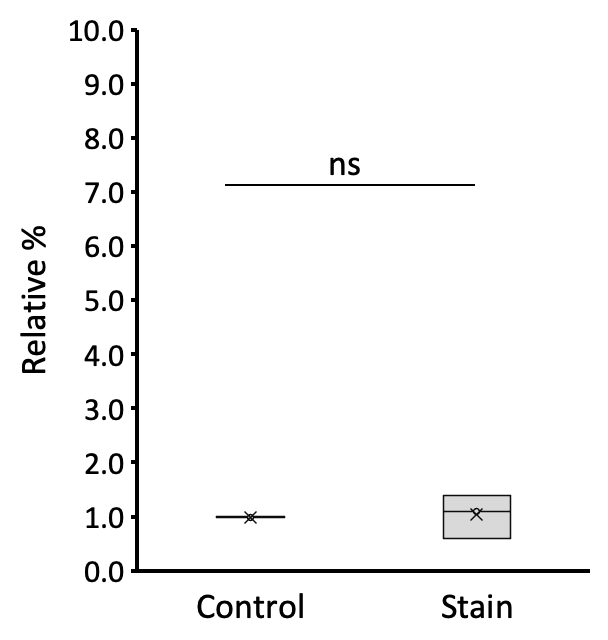


**Figure S3.** Bar plot showing the ratio of cells undergoing tripolar mitosis versus bipolar mitosis under two conditions: Control (no dyes added) and Stain (both SPY650 DNA dye and photoactivatable dye added). ~2,700-2,900 mitotic events were analyzed in both conditions (N = 3). The p-value was obtained using Student’s t-test. ns (not significant).


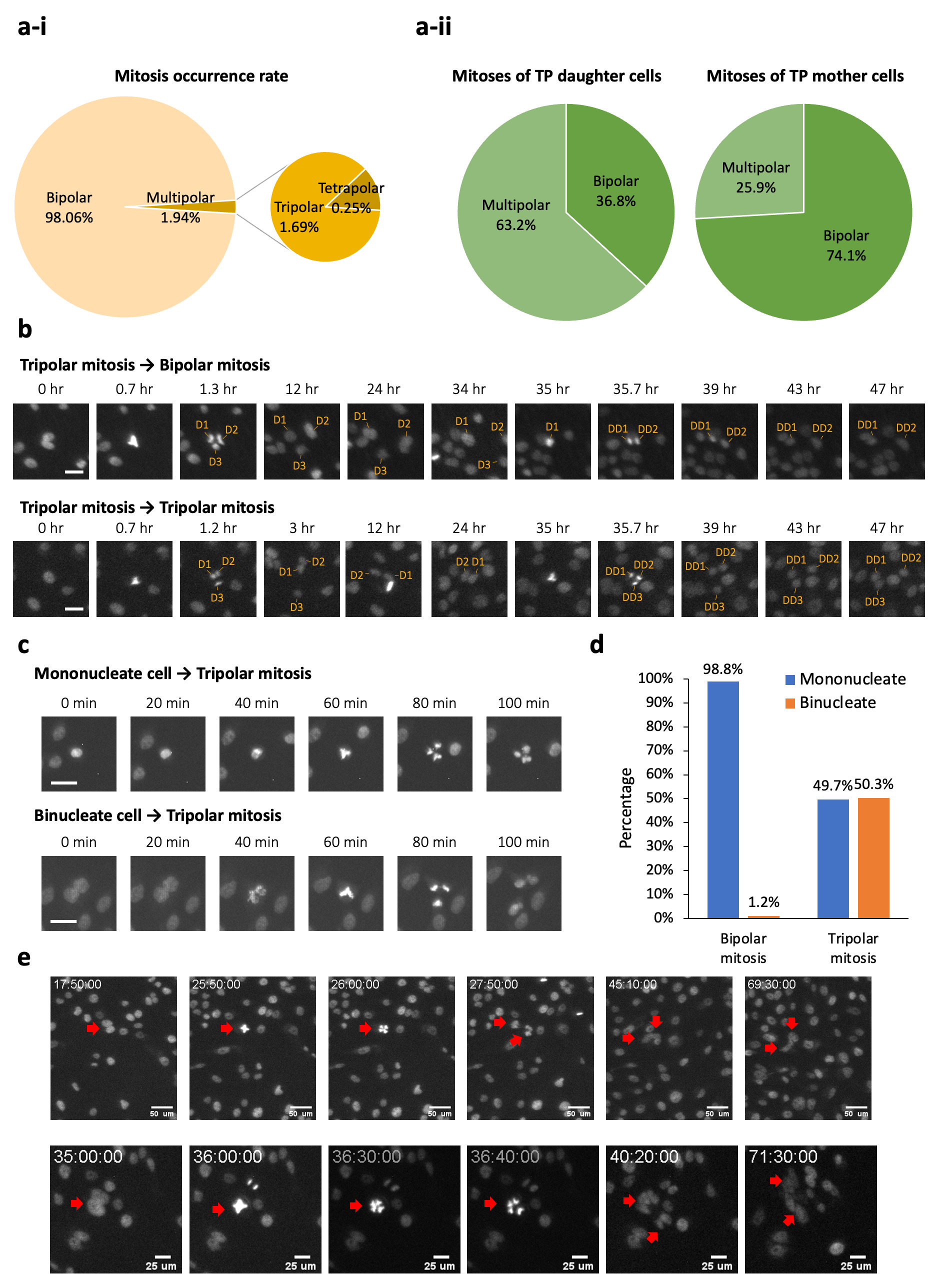


**Figure S4. Quantification of multipolar mitoses in MCF10A cells**. a-i) Statistics of the rates of bipolar and multipolar mitoses in MCF10A cells. a-ii) Statistics of mitosis phenotypes for TP daughter cells and mother cells. b) Representative time-lapse images of tripolar daughter cell(s) followed by either bipolar mitosis (upper panels) or tripolar mitosis (lower panels). c) Representative time-lapse images of tripolar daughter cells dividing from mononucleate cells (upper panels) or binucleate cells (lower panels). d) Statistics of the rates of bipolar or tripolar mitoses originating from mononucleate cells or binucleate cells. All the scale bars denote 30 μm. e) Two representative cases (upper and lower panels) of tetrapolar mitosis over time.


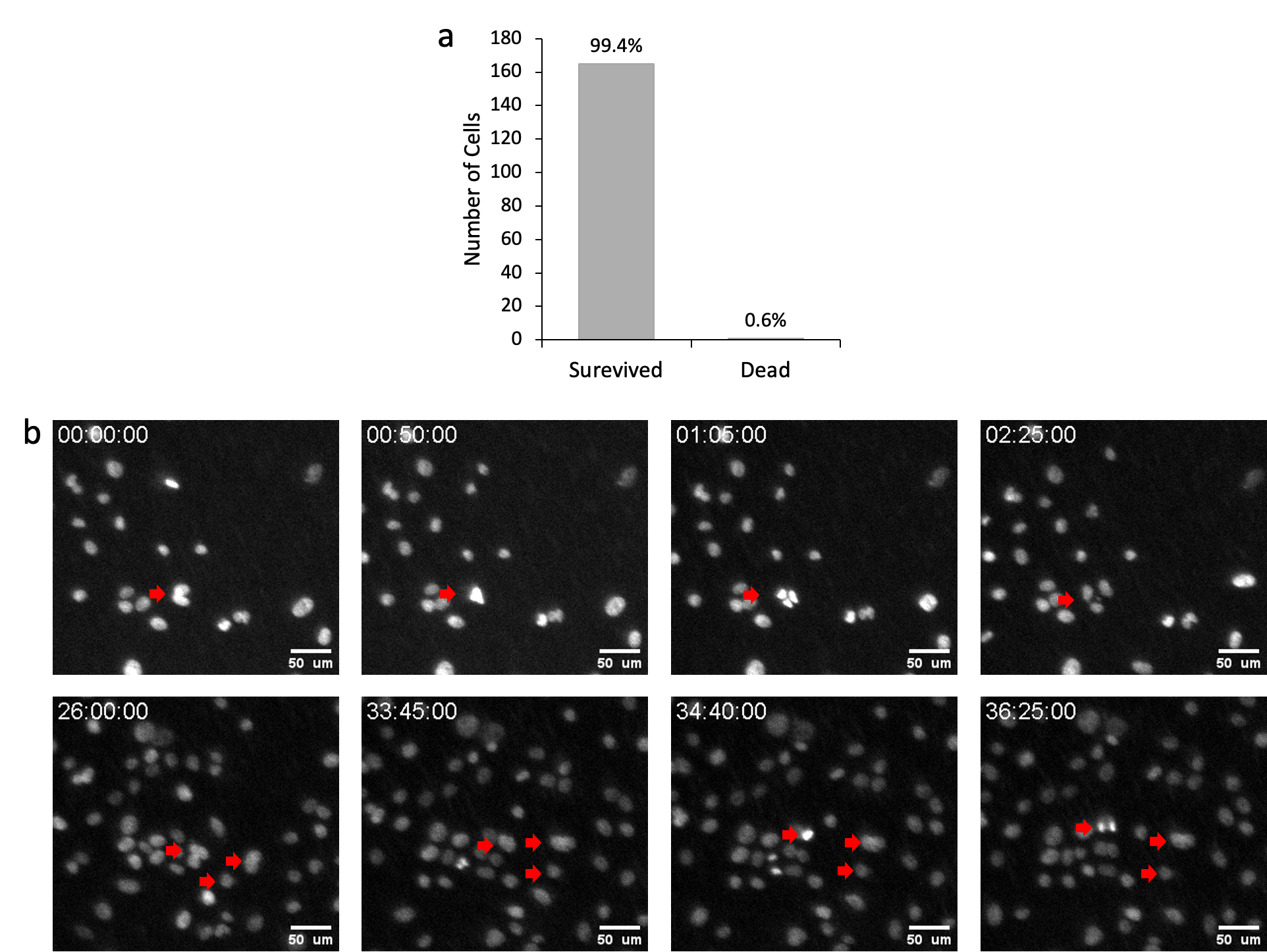


**Figure S5. Survival rate of cells after tripolar mitosis (TP cells)**. a) A total of 166 TP cells were monitored for 24 hours or until the next mitosis; of these, 165 cells survived and 1 cell died. b) Time-course images showing a representative case of tripolar mitosis and the cell fate of the three daughter cells. One of them subsequently underwent bipolar mitosis at 36.5 hours. The scale bars denote 50 µm.


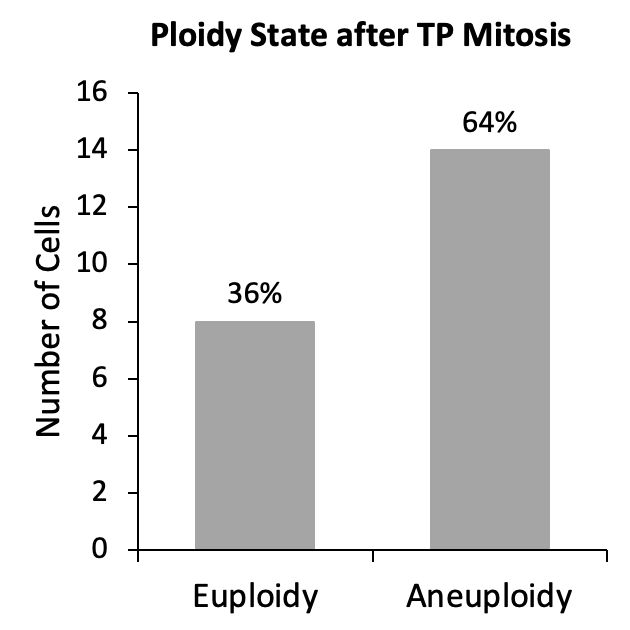


**Figure S6**. Bar plot showing the number of euploidy or aneuploidy cells after tripolar mitosis.


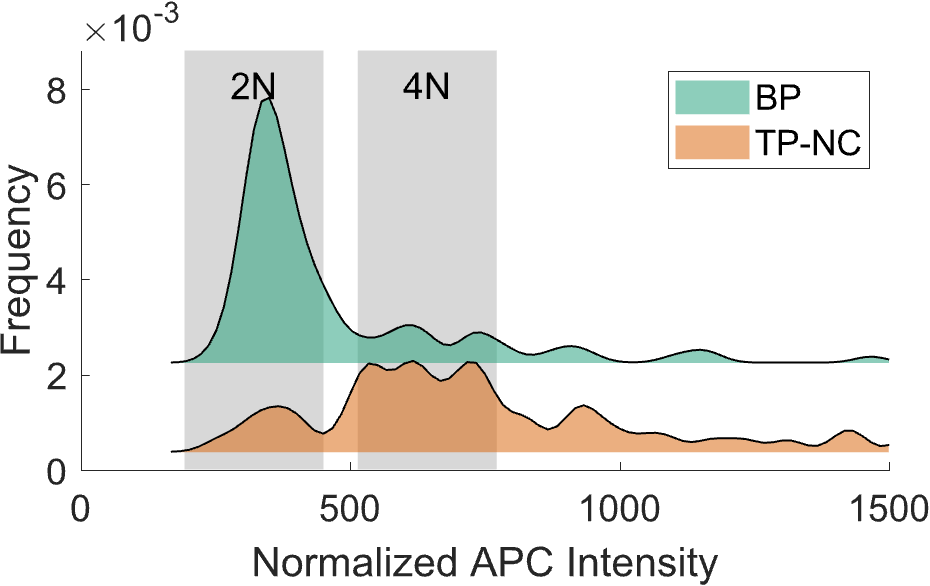


**Figure S7**. Flow cytometry plots show the DNA content (indicated by APC intensity) of BP and TP-NC cells.

**Figure S8. Validation of polyploidy in TP-NC cells**. a) Plot of nuclei fusion status, acquired from live-cell time-lapse imaging, within a single cell after tripolar mitosis. ~65% of cases displayed 2-nuclei fusion, with 2 nuclei aggregating within a single cell, while ~30% of cases exhibited 3-nuclei fusion, with 3 nuclei merging within a single cell, after tripolar mitosis. b) Representative time-lapse images (bright field (lower panel) and SPY650-DNA nuclear staining (upper panel)) depict cells undergoing 3-nuclei fusion after tripolar mitosis (red asterisk)). Scale bars denote 10 μm. c) FISH assay targeting Chromosome 6 (Chr 6) on fixed cells, corresponding to the last panel of figure e, wherein Chr 6 is visualized in red foci. White dashed lines delineate cell membrane boundaries. The scale bar denotes 10 μm.


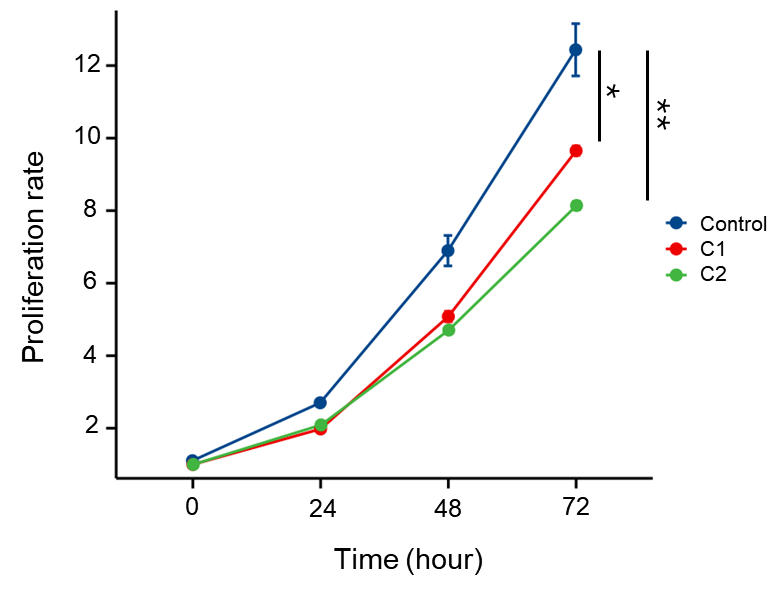


**Figure S9. Cell proliferation rate of the bipolar and tripolar cell clones.** Cell proliferation rates of the Control bipolar cell clone (blue) and two tripolar cell clones, C1 (red) and C2 (green), were measured at 0, 24, 48, and 72 hours using the CCK-8 assay. Absorbance values were normalized. The proliferation rate increased over time in all groups but was significantly higher in the Control group compared to C1 (p < 0.05 *) and C2 (p < 0.01 **). The p-values were obtained using Two-way repeated-measures ANOVA test.


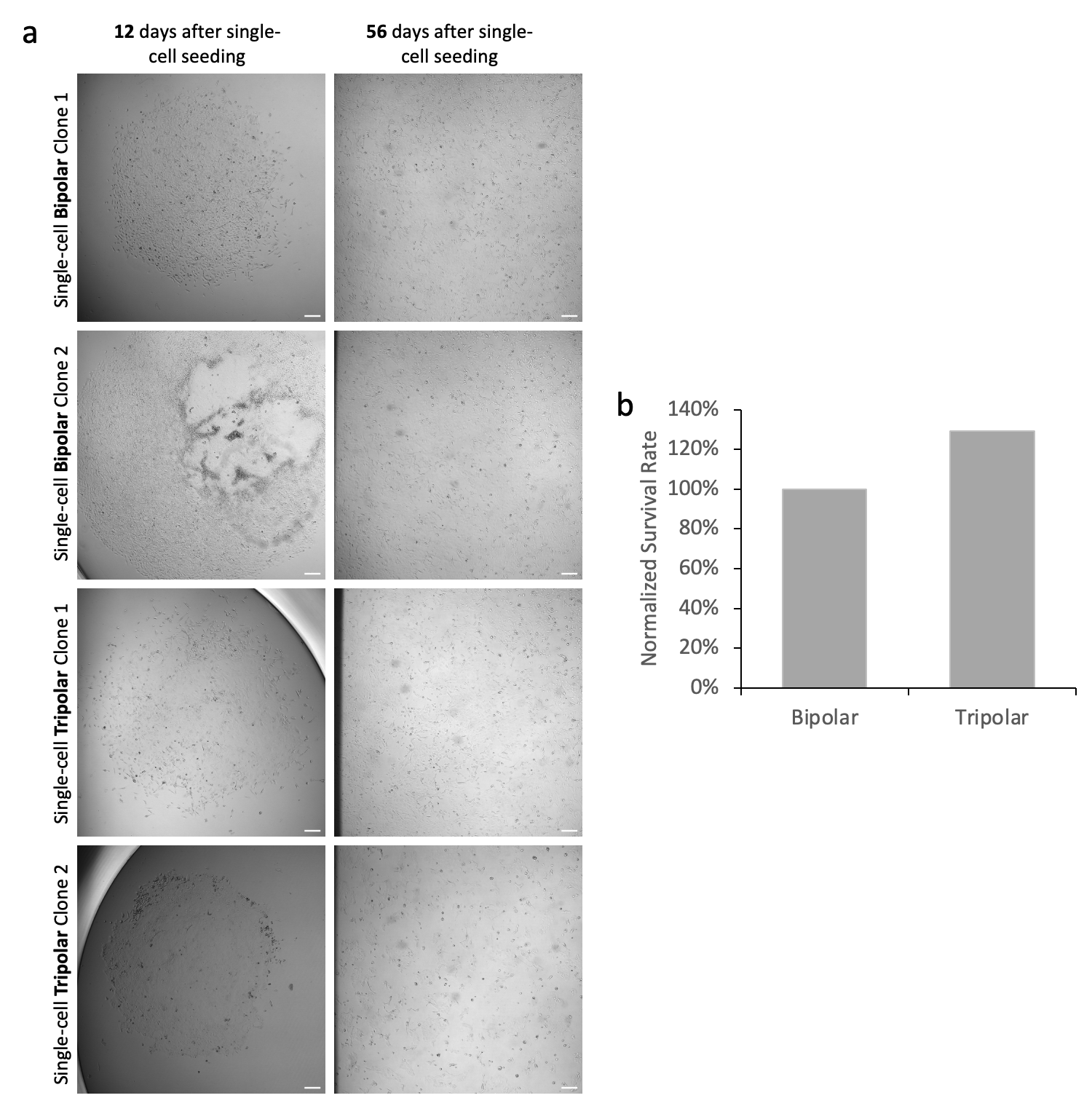


**Figure S10**. **Clonogenic assay**. a) Two representative single-cell clones following either bipolar or tripolar mitosis. Images were taken on Days 12 and 56 for these single-cell clones. Scale bars, 200 µm. b) Normalized survival rate (relative to the survival rate of bipolar cell clones). 30 single-cell bipolar clones and 116 single-cell tripolar clones were collected for the assay.


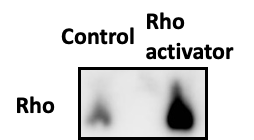


**Figure S11. Western blot showing Rho protein expression following Rho perturbation.** Western blot analysis showing Rho activation in cells treated with a Rho activator compared with the control group.

**Figure S12**. **The top 20 differential genes between different cell subclusters (BP-S0, BP-S1, TP-S1, and TP-S2) related to the degranulation-like pathway are shown**. Genes related to enzymes or proteases are highlighted in red. The BCL2L1 gene is shown in blue.


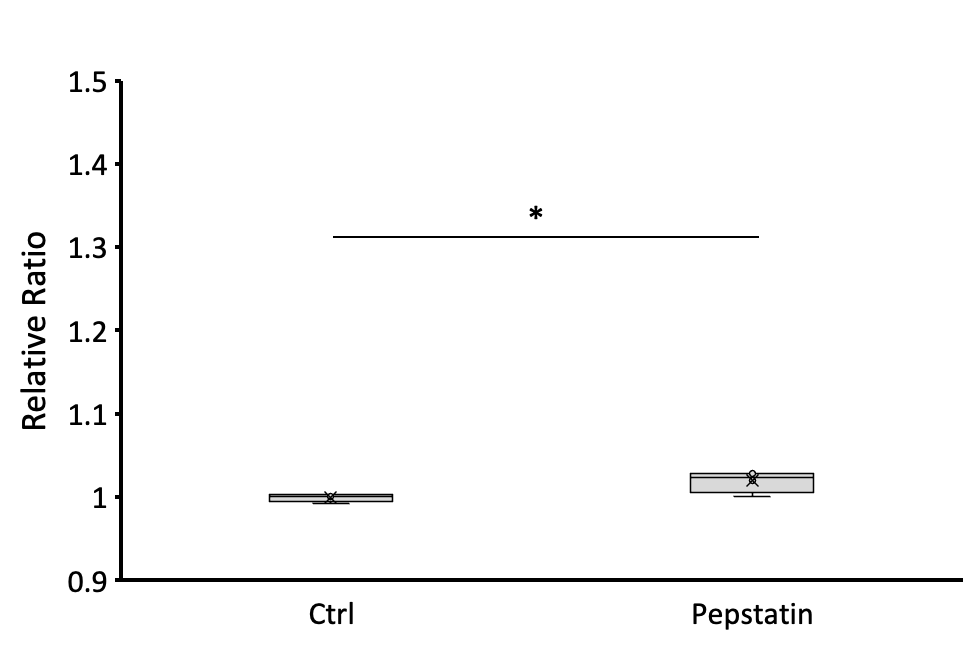


**Figure S13**. Relative survival rate ratios of TP cells under Control (Ctrl) and Pepstatin-treated, conditions (N = 5). The p-values were calculated using Student’s t-test. p < 0.05 *.


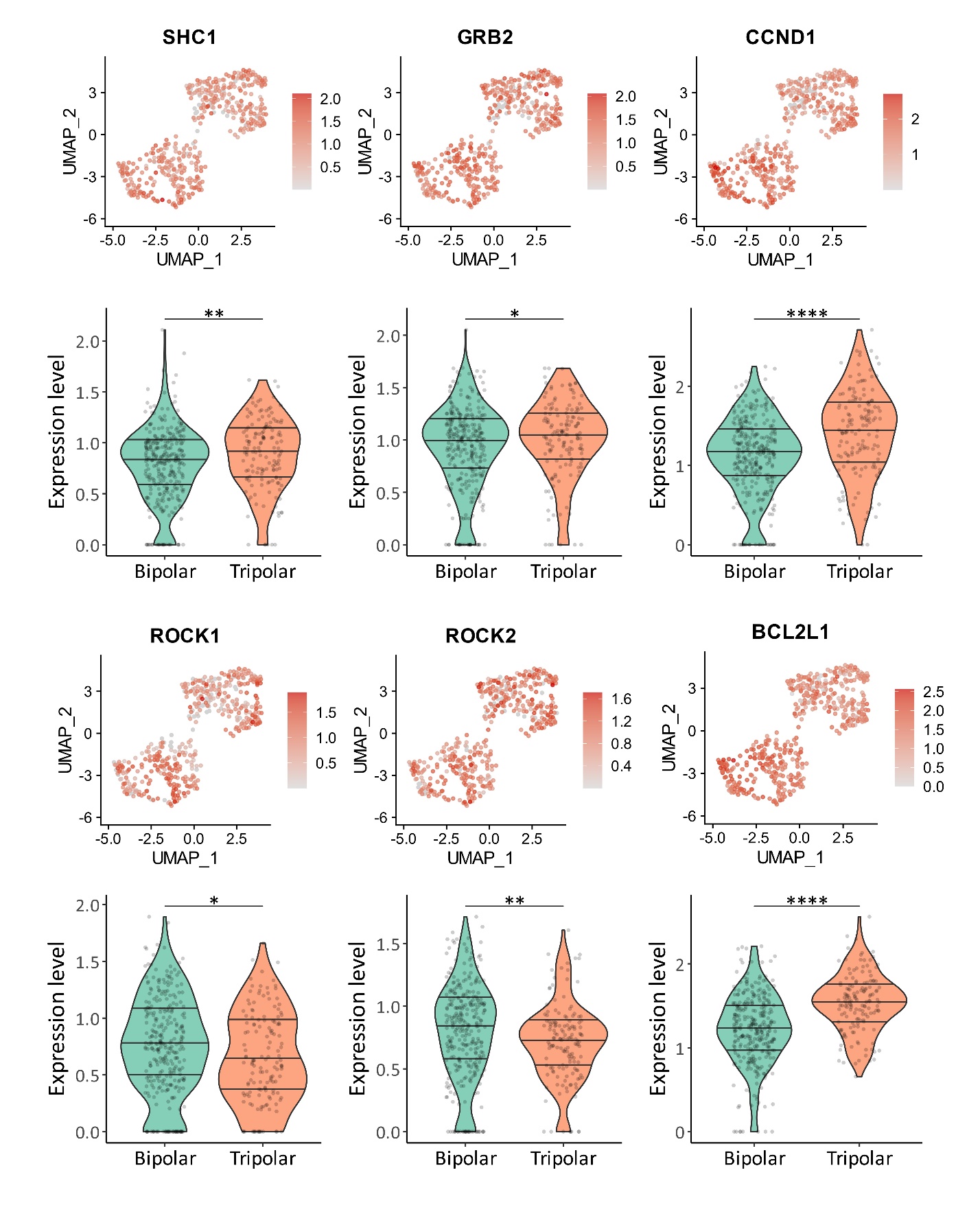


**Figure S14**. **UMAP plots (upper panels) and violin plots (lower panels) display the module score of genes associated with the PTEN signaling pathway**, specifically SHC1, GRB2, CCND1, ROCK1, ROCK2, and BCL2L1 genes, for bipolar and tripolar cells. The p-value was obtained using Wilcoxon test. Bonferroni correction; p < 0.05 * ; p < 0.01 **; p < 0.001 ***; p < 0.0001 ****. The overlaid quantiles (25th, 50th, 75th percentiles) represent the data spread. Individual data points are shown as jittered points. Statistical analysis was carried out using R Software.


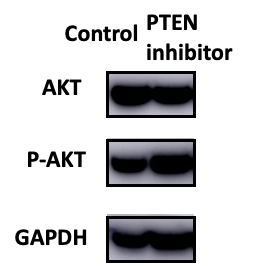


**Figure S15. Western blots showing AKT protein expression and phosphorylated AKT following PTEN inhibition**. Western blot showing the effects of PTEN inhibition on AKT signaling. Treatment with a PTEN inhibitor increased AKT phosphorylation (p-AKT) without altering total AKT levels. GAPDH was used as a loading control. Please note that PTEN inhibition enhances AKT phosphorylation, while increased PTEN expression suppresses phosphorylated AKT levels.


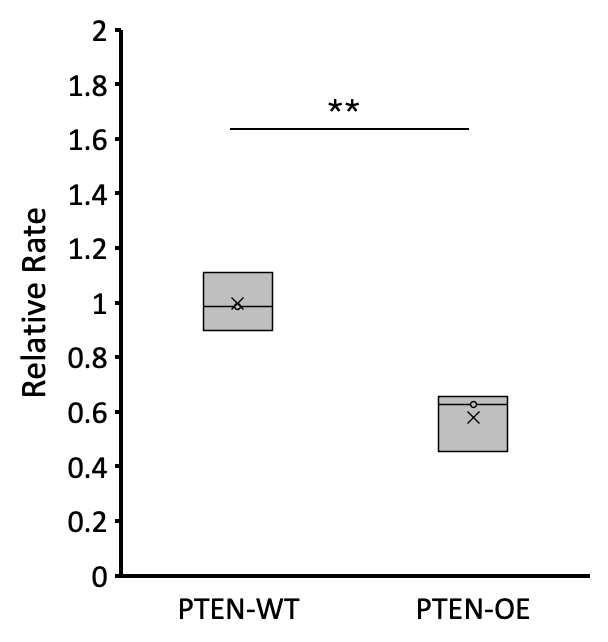


**Figure S16**. Box plot shows the normalized tripolar mitosis rate ratios of the Control (PTEN-wild type (WT)) and PTEN-overexpression (PTEN-OE) groups (N = 3, Student’s t-test).


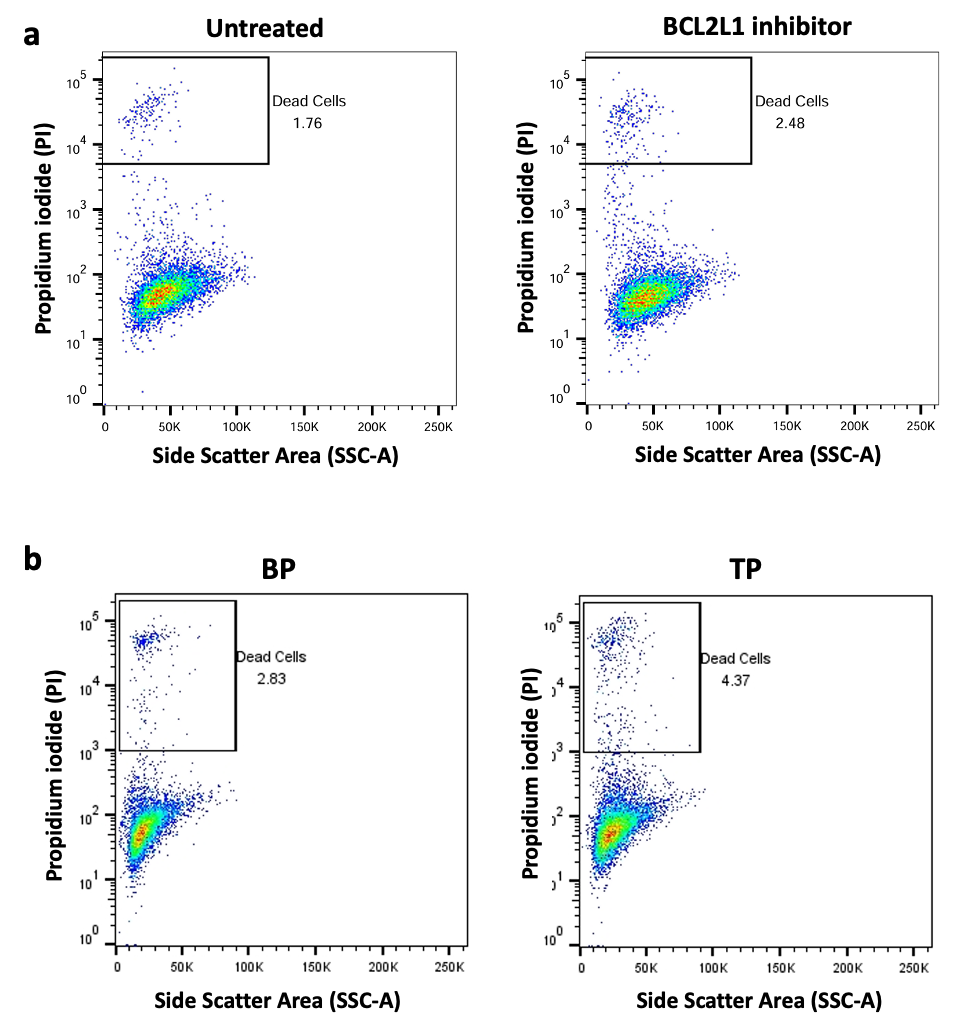


**Figure S17**. **Flow cytometry plots of MCF10A cells following BCL2L1 inhibition**. a) left: untreated control; right: cells treated with 0.5 nM BCL2L1 inhibitor (A-1331852; Tocris Bioscience, #7661) for 4 hours. b) Flow cytometry plots of a BP cell clone (left) and a TP cell clone (right) treated with 0.5 nM BCL2L1 inhibitor (A-1331852; Tocris Bioscience, #7661) for four hours. The x-axis represents Side Scatter Area (SSC-A), and the y-axis represents Propidium Iodide (PI) fluorescence, a cell death indicator.


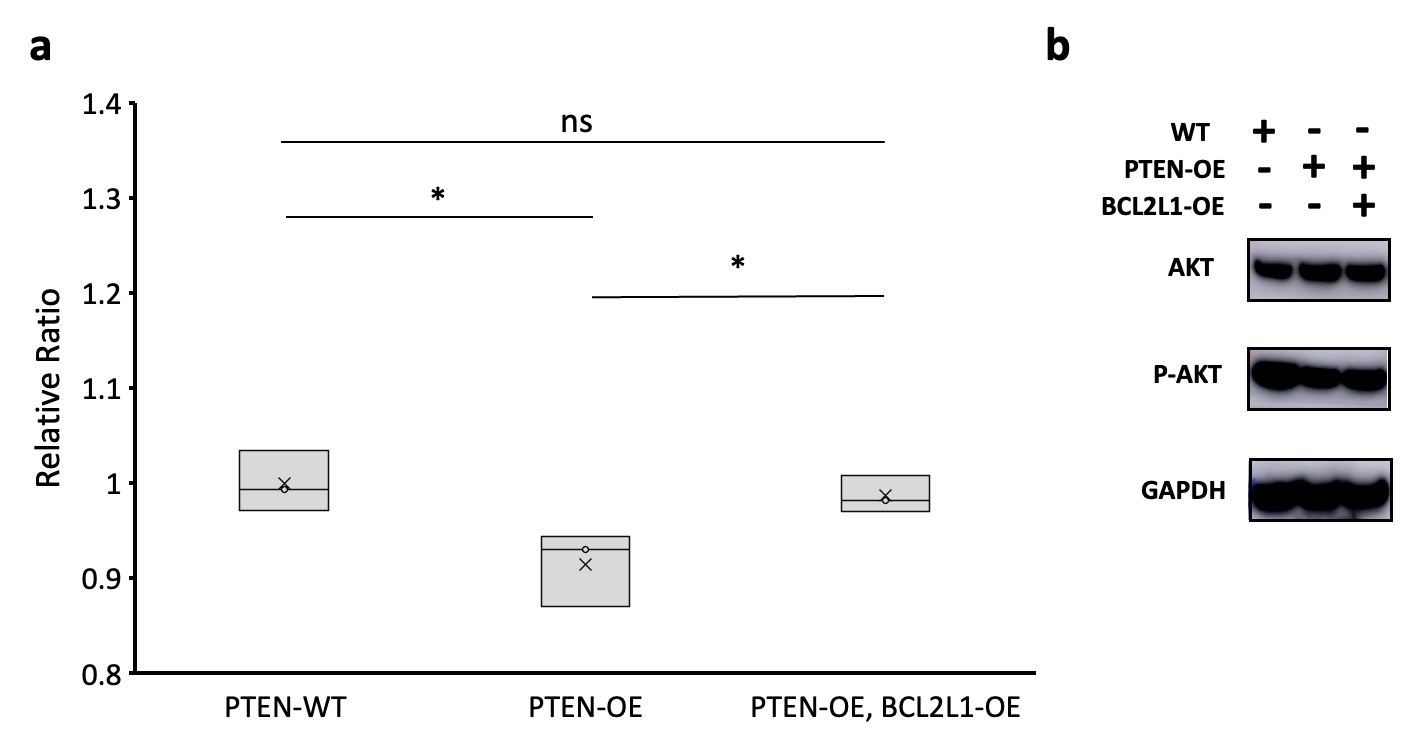


**Figure S18**. **Validation assays with PTEN and BCL2L1 perturbation**. a) Box plot shows the normalized survival rate ratios of TP cells in the Control, PTEN-overexpression (OE), and PTEN-OE/BCL2L1-OE groups (N = 3). The p-values were calculated using Student’s t-test. ns (not significant); p < 0.05 *. b) Western blot analysis shows total and phosphorylated AKT (p-AKT; a functional indicator of PTEN inhibition) levels in cells overexpressing PTEN (PTEN-OE) or co-overexpressing PTEN and BCL2L1 (BCL2L1-OE). Overexpression of PTEN decreased AKT phosphorylation, whereas co-overexpression of BCL2L1 partially restored p-AKT levels. GAPDH was used as a loading control. Please note that PTEN inhibition enhances AKT phosphorylation, while increased PTEN expression suppresses phosphorylated AKT levels.


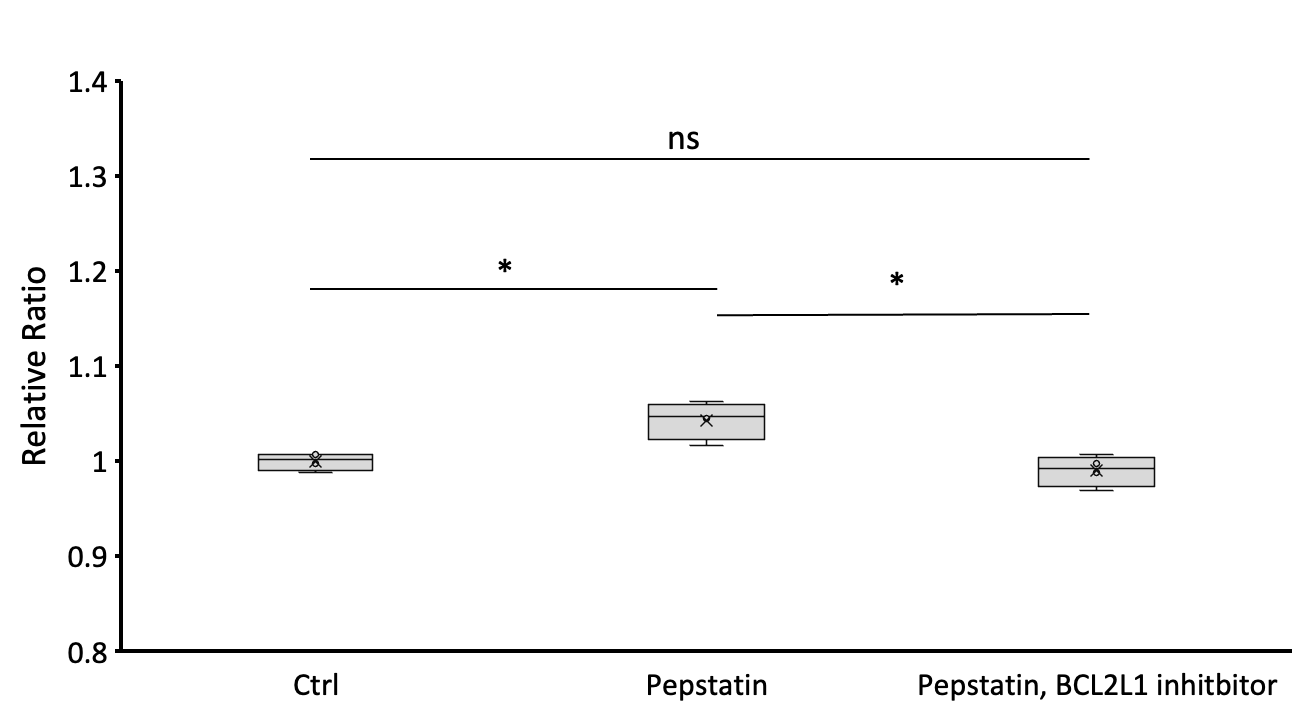


**Figure S19**. Relative survival rate ratios of TP cells under Control (Ctrl), Pepstatin-treated, and Pepstatin plus BCL2L1 inhibitor co-treated conditions (N = 4). P-values were calculated using Student’s *t*-test. ns (not significant); p < 0.05 *.


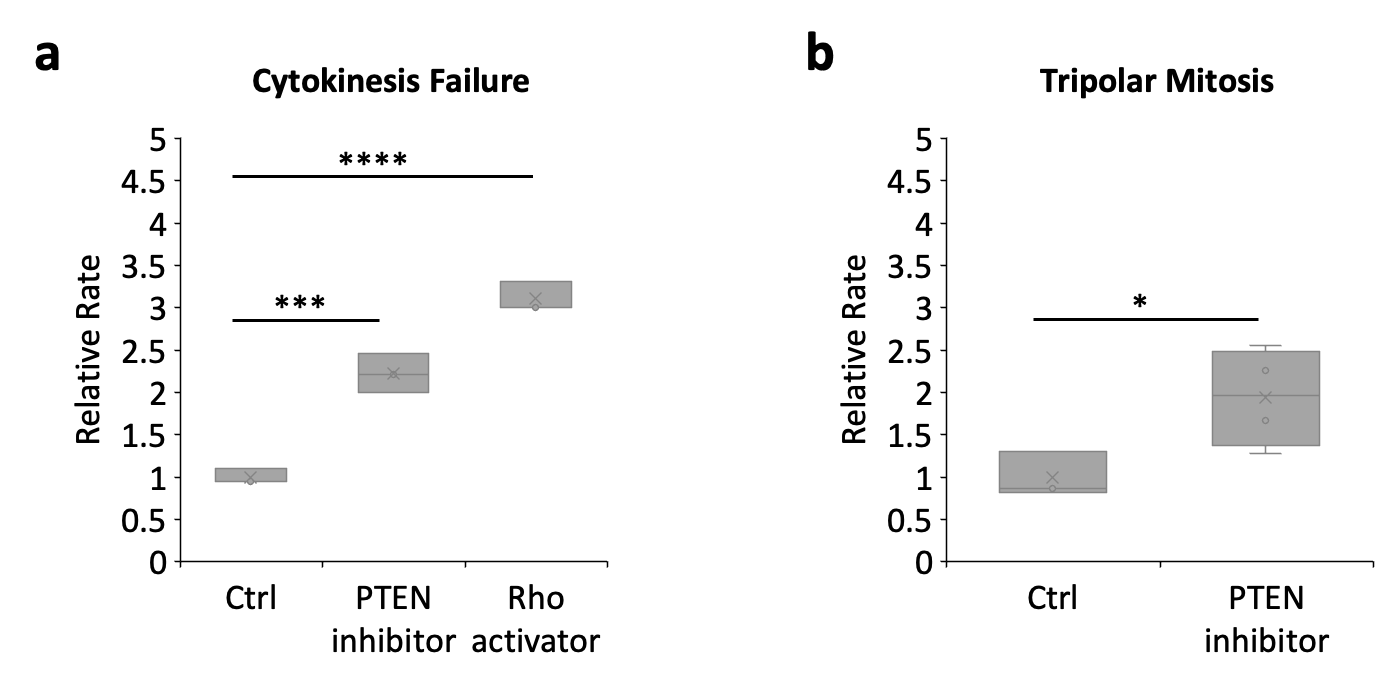


**Figure S20.** **Upregulation of RhoGTPase signaling and downregulation of PTEN in MCF7 cells**. a) Statistics of cytokinesis failure rate in MCF7 cells treated without drugs (Control, Ctrl), with a PTEN inhibitor, or with a RhoGTPase activator (N = 3, Student’s t-test). b) Statistics of tripolar mitosis rate in MCF7 cells treated without drugs (Control, Ctrl) or with a PTEN inhibitor (N = 3). The p-value was obtained using Student’s t-test**.** In the box and whisker plots, the box shows the interquartile range (25th, 50th, 75th percentiles), and whiskers indicate the minimum and maximum values. The p-value was obtained using Student’s t-test. ns (not significant); p < 0.05 *; p < 0.01 **; p < 0.001 ***; p < 0.0001 ****.


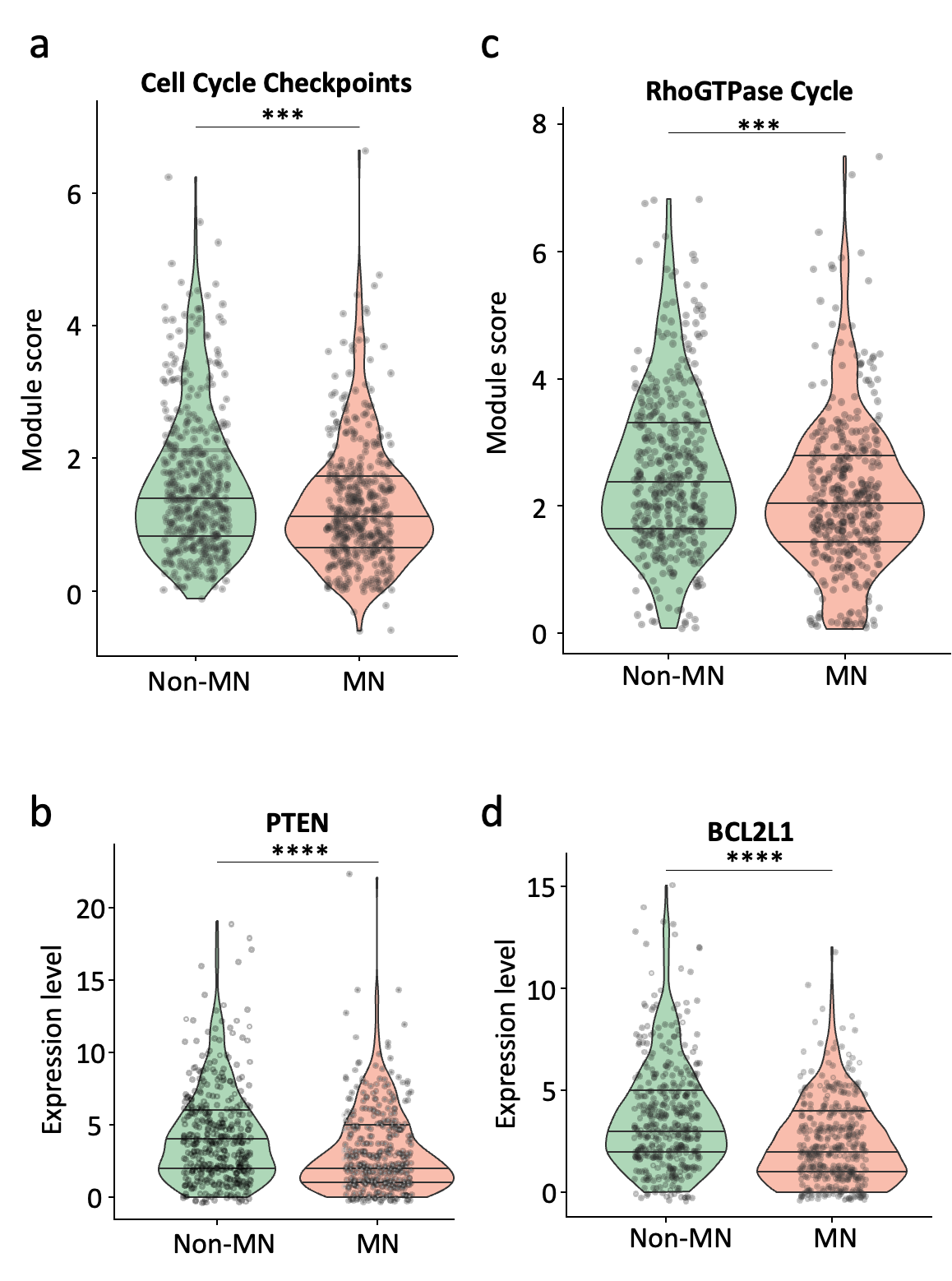


**Figure S21**. a) A violin plot shows the module score of the Cell Cycle Checkpoints pathway for non-micronucleated (Non-MN) and micronucleated (MN) HeLa cells. b) A violin plot shows the expression of the PTEN gene for Non-MN and MN HeLa cells. c) A violin plot presents the module score of the RhoGTPase Cycle pathway for non-MN and MN HeLa cells. d) A violin plot shows the expression of the BCL2L1 gene for Non-MN and MN HeLa cells. The p-value was obtained using Wilcoxon test with Bonferroni correction. p < 0.001 ***; p < 0.0001 ****. The overlaid quantiles (25th, 50th, 75th percentiles) represent the data spread. Individual data points are shown as jittered points.
